# Role of Rilpivirine and Etravirine in Efavirenz and Nevirapine-Based Regimens Failure in a Resource-Limited Country: A Cross- Sectional Study

**DOI:** 10.1371/journal.pone.0154221

**Published:** 2016-04-27

**Authors:** Phairote Teeranaipong, Sunee Sirivichayakul, Suwanna Mekprasan, Pirapon June Ohata, Anchalee Avihingsanon, Kiat Ruxrungtham, Opass Putcharoen

**Affiliations:** 1 Department of Parasitology, Faculty of Medicine, Chulalongkorn University, Bangkok, Thailand; 2 Division of Allergy and Clinical Immunology, Department of Medicine, Faculty of Medicine, Chulalongkorn University, Bangkok, Thailand; 3 HIV Netherlands Australia Thailand Research Collaboration, The Thai Red Cross AIDS Research Centre, Pathumwan, Bangkok, Thailand; 4 Division of Infectious Diseases, Department of Medicine, Faculty of Medicine, Chulalongkorn University, Bangkok, Thailand; University of Malaya, MALAYSIA

## Abstract

**Introduction:**

Etravirine(ETR) can be used for patients who have failed NNRTI-based regimen. In Thailand, ETR is approximately 45 times more expensive than rilpivirine(RPV). However, there are no data of RPV use in NNRTI failure. Therefore, we assessed the susceptibility and mutation patterns of first line NNRTI failure and the possibility of using RPV compared to ETV in patients who have failed efavirenz(EFV)- and nevirapine(NVP)-based regimens.

**Methods:**

Clinical samples with confirmed virological failure from EFV- or NVP-based regimens were retrospectively analyzed. Resistance-associated mutations (RAMs) were interpreted by IAS-USA Drug Resistance Mutations. Susceptibility of ETR and RPV were interpreted by DUET, Monogram scoring system, and Stanford University HIV Drug Resistance Database.

**Results:**

1,279 and 528 patients failed EFV- and NVP-based regimens, respectively. Y181C was the most common NVP-associated RAM (54.3% vs. 14.7%, p<0.01). K103N was the most common EFV-associated RAM (56.5% vs. 19.1%, P<0.01). The results from all three scoring systems were concordant. 165(11.1%) and 161(10.9%) patients who failed NVP-based regimen were susceptible to ETR and RPV, respectively (p = 0.85). 195 (32.2%) and 191 (31.6%) patients who failed EFV-based regimen, were susceptible to ETR and RPV, respectively (p = 0.79). The susceptibility of ETV and RPV in EFV failure was significantly higher than NVP failure (p<0.01).

**Conclusion:**

The mutation patterns for ETR and RPV were similar but 32% and 11% of patients who failed EFV and NVP -based regimen, respectivly were susceptible to RPV. This finding suggests that RPV can be used as the alternative antiretroviral agent in patients who have failed EFV-based regimen.

## Introduction

Recently, the guideline for the use of antiretroviral (ARV) agents in HIV-1-Infected adults and adolescents[1] has recommended two nucleotide reverse transcriptase inhibitors (NRTIs) in combination with another active ARV drug from an integrase strand transfer inhibitor (INSTI) or a protease inhibitor (PI) with a pharmacokinetic enhancer (cobicistat or ritonavir) for treatment-naïve HIV-1-infected patients, while a non-nucleoside reverse transcriptase inhibitor (NNRTI)-based regimen has become the “alternative regimen” because of its side effects and low genetic barrier. However, in many resource-limited countries, including Thailand, the NNRTI-based regimen is widely used and is currently recommended by WHO (World Health Organization) and the Thai national guidelines[2,3] as the initial regimen for HIV-1 treatment-naïve adult and adolescent patients. For patients who developed virological failure, the second-line regimen should be carefully designed to ensure long-term efficacy. The consideration for new regimen is largely based on the viral resistance profile and most importantly, financial affordability of the subsequent regimen.

The cost of the medications usually will dictate the availability of ARVs, particularly second-line antiretroviral agents. In many developing countries, including Thailand, efavirenz (EFV) is the most preferred NNRTI for the first-line regimen which is also in line with the current WHO’s recommendation for the treatment of HIV-infected patients because of its potency and affordability. The other alternative choice for patients who cannot tolerate the side effects of EFV is nevirapine (NVP) but one of its limiting factor is that it can only be used in HIV-infected patients with low CD4 level.

The list of available NNRTIs has now expanded to include second-generation NNRTIs such as rilpivirine (RPV) and etravirine (ETR). RPV is recently recommended and approved to be used in combination with two NRTIs for treatment-naïve adult patients with pre-treatment HIV-1 RNA < 100,000 copies/ml[4–7] and pre-treatment CD4 counts of more than 200 cells/μl[6,7]. Another factor that may be of concern is its cross-resistance to all NNRTIs, especially with NVP[5,8–11], so extreme caution is warranted when choosing RPV as the patient’s initial regimen.

On the other hand, there are some data suggests that a presence of single NNRTI resistance­associated mutations (RAMs) cannot significantly render RPV inactive. As a matter of fact, it would require a total of 8 changes at the HIV­1 reverse transcriptase gene to severely lower the susceptibility of RPV. According to the ECHO and THRIVE studies, E138K was the most frequently selected mutation (45%) detected in ARV­naive patients who have failed RPV therapy which is also often seen with M184I (34%), confering resistance to both lamivudine (3TC) and emtricitabine (FTC)[6,7].

In contrast, the other second generation NNRTI, etravirine (ETR), is highly efficacious when used in treatment-experienced patients with boosted-darunavir (DRV/R) and thus is approved for use in these types of patients[12,13]. The chemical structure of ETR is similar to that of RPV, yet the latter is approved for use only in treatment-naive patients and have not been investigated in treatment-experienced patients because the reports from the ECHO and THRIVE studies have precluded its further investigation into its use in ART-resistant patients.

Because of this, RPV looks very attractive as a possible second-line ARV agent for Thailand which has approximately 1 million HIV-infected people. In 2014, 0.3 million HIV-infected patients were treated with antiretroviral therapy (ART) through the National AIDS Treatment Programme of which approximately 15,000 (5%) were on second-line ART and 500 patients on third-line ART. It is assumed that every year, a certain number of HIV-infected patients will fail their ART regimens and may transmit drug-resistant viruses to other high-risk populations[14,15]. With time and increased number of patients that will fail the first-line ART regimens, soon there will be a need for second-line ART. However, the cost of a second-line regimen represents a major challenge because it is approximately 10–15 times more expensive compared to the first-line ART regimen.

Hence, for resource-limited setting, alternative strategies have to be explored before resorting to using extravagant second-line regimen. At the time of writing this manuscript, the National Health Security Office (NHSO) is considering to include second generation NNRTI (RPV) in its essential list of drugs to be covered by the national program which is more affordable compared to the recommended ARV agents such as PI or INSTI or other second generation NNRTI (i.e., ETR) for the second-line ART regimen. The strategy of using RPV in patients who cannot afford or tolerate ETR is the driving force for this study because there is very limited data whether RPV can be safely used in patients with NVP or EFV failure. We therefore investigated the role of RPV compared to ETR, as the second-line ARV agent for HIV-1 infected Thai patients who experienced treatment failure from NVP- or EFV-based regimens.

The findings from this study will provide invaluable information on the susceptibility and mutation patterns of RPV in patients who failed first-generation NNRTI regimens so the patients’ subsequent regimens can appropriately be selected. For resource-limited countries, a cheaper alternative choice for second-line regimen such as RPV might be extremely beneficial. Not only will this provide treatment-experienced patients with wider access to RPV but also help improve their adherence.

## Methods

The study was approved by the Institutional Review Board of Faculty of Medicine, Chulalongkorn University, Bangkok, Thailand. All written informed consents were obtained from participants prior to any procedures.

### Study population

This cross-sectional study collected data from HIV-NAT, King Chulalongkorn Memorial Hospital, and its network hospitals in Bangkok from January 2003 to November 2010. Resistant testings for ETR and RPV were done for HIV-1-infected adults (more than 16 years of age) with HIV-1 viral load more than 1,000 copies/ml and have failed either EFV- or NVP-based regimen.

### Detection of mutations at the HIV-1 reverse transcriptase (RT) location and analysis of the genotypic resistance of ETR and RPV

Viral RNA was extracted from freshly thawed patient’s plasma. RT-PCR for HIV-1 *RT* gene was performed as previously described [16]. Mutations were examined by direct sequencing. RT-RAMs were identified and analyzed by using the Stanford Drug Resistance Database for V90I, A98G, L100I/V, K101E/P/Q/H/N, K103N/S/T/Q/E/H/R, V106A/M/I, V108I, E138A/K/Q/G/R, V179D/E/T/F/L, Y181C/I/V/S/F/G, M184I, Y188C/H/L/F, G190A/S/E/Q/C/V/T, H221Y, P225H, F227C/L, M230L/I, P236L, K238T/N, Y318F and N348I. NNRTI susceptibility/resistance interpretation was obtained from the HIV Database Program Genotypic Resistance Interpretation Algorithm version a7.0, last updated February 28, 2014 (http://hivdb.stanford.edu)[17]. In brief, the algorithm correlated a score to each single mutation and the weight of a specific combination of synergistic mutations is based on the new, updated, universal weighted list of mutations. The total mutation penalty score was defined as the level of susceptibility/resistance to each NNRTI according to the following ranges: score between 0–9 suggests that the drug is still genotypically susceptible because the susceptibility of the drug did not decrease even in the presence of the wild-type virus, score between 10–14 suggests existence of potentially low-level resistance, score between 15–29 suggests that there is low-level resistance, score between 30–59 suggests that there is an intermediate resistance, and a score of 60 or more indicated the presence of high-level resistance. Samples from patients that have high scores of resistance to NVP- or EFV-based regimen were selected and analyzed.

### Statistical analysis

The number of NNRTI-RAMs per person and the differences of the distribution of NNRTI-RAMs for either EFV- or NVP-based regimen were analysed by Pearson’s chi-squared test. Susceptibility/resistance analysis within EFV- or NVP-based regimen (paired samples; susceptibility of ETR vs. RPV in failed EFV- or NVP-based regimen) was analyzed by using Wilcoxon signed-rank test. Pearson’s correlation coefficients were calculated. A p-value of less than 0.05 was considered statistically significant.

## Results

**Fig 1:**
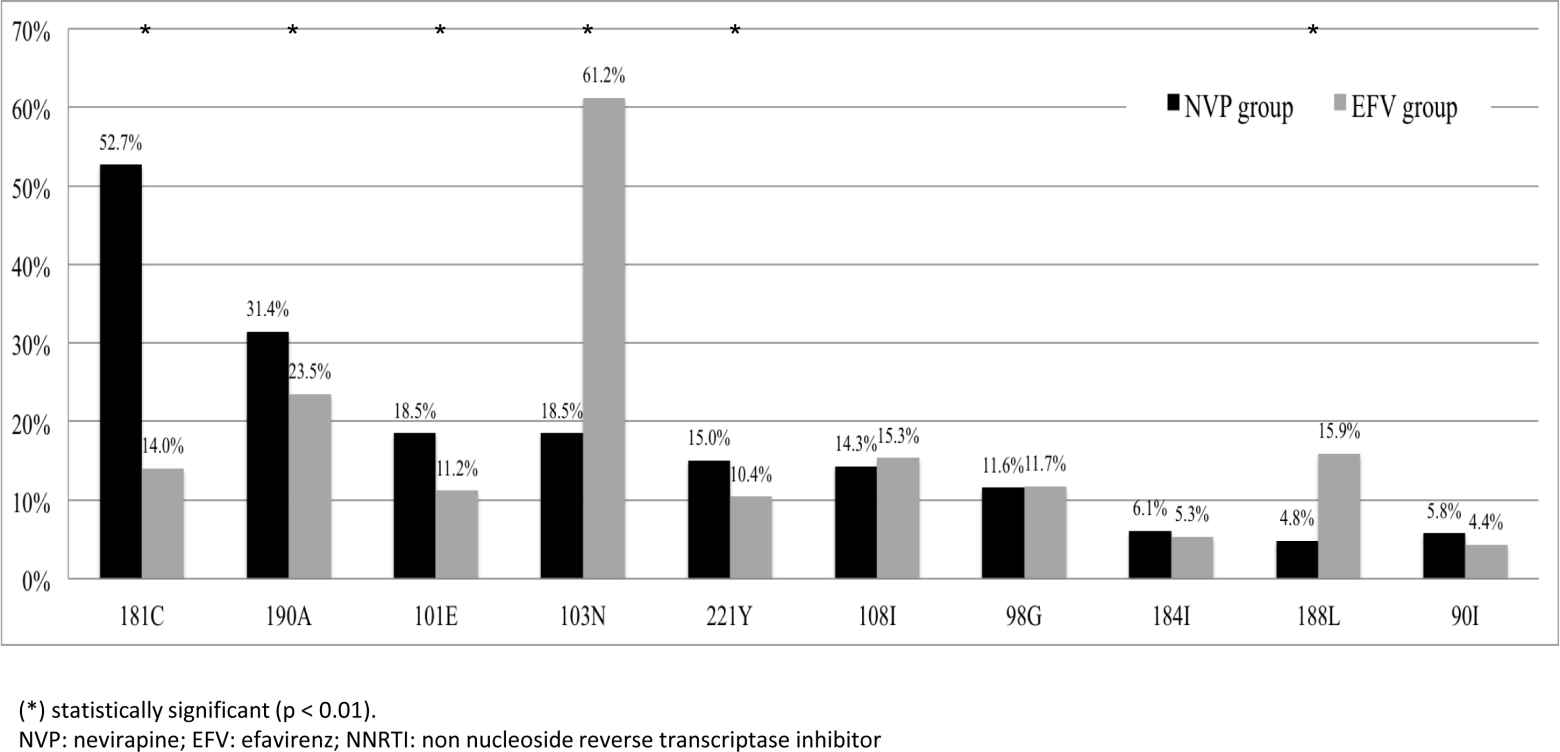
Top 10 NNRTI-RAMs found in patients who failed either the NVP- or EFV-based regimen.

A total of 1,807 patients were included in this analysis. There were 1,279 patients that failed the NVP-based regimen and another 528 patients that failed the EFV-based regimen. Baseline characteristics of the patients are shown in Table 1. The median for plasma viral load for patients who failed NVP- and EFV-based regimens were 12,500 and 11,373 copies/ml, respectively (p = 0.18). The median CD4 cell count at time of failure for patients who failed NVP- and EFV-based regimens were 170 and 130 cells/μl, respectively (p = 0.23). The NRTIs used in the NVP-based regimen were 3TC (96.5%), stavudine (d4T) (76.9%) and zidovudine (AZT) (19.2%). The NRTIs used in the EFV-based regimen were 3TC (83.9%), d4T (38.8%) and AZT (42.4%) (Fig 1). A small proportion of patients who failed NVP- and EFV-based regimens were on didanosine (DDI), abacavir (ABC) and tenofovir disoproxil fumarate (TDF). The most common NRTI backbones used were AZT + 3TC, and d4T + 3TC.

**Table 1:** Baseline characteristics of the patients.

Characteristics	NVP failure	EFV failure	p value
Number of patients	1,279	528	
CD4 count, median (cells/ul)	170	130	0.23
Plasma HIV viral load, median (copies/ml)	12,500	11,373	0.18
ARV of choice			
- zidovudine	245 (19.2%)	224 (42.4%)	< 0.01
- stavudine	984 (76.9%)	205 (38.8%)	< 0.01
- didanosine	63 (4.9%)	80 (15.2%)	< 0.01
- abacavir	5 (0.4%)	13 (2.5%)	< 0.01
- lamivudine	1,234 (96.5%)	443 (83.9%)	< 0.01
- tenofovir	21 (1.6%)	60 (11.4%)	< 0.01
NRTI combination			
- zidovudine + lamivudine	209 (16.3%)	179 (33.9%)	< 0.01
- stavudine + lamivudine	956 (74.7%)	166 (31.4%)	< 0.01
- tenofovir + lamivudine	17 (1.3%)	54 (10.2%)	< 0.01
- zidovudine + didanosine	20 (1.6%)	33 (6.3%)	< 0.01
- stavudine + didanosine	10 (0.8%)	24 (4.5%)	< 0.01
- didanosine + lamivudine	24 (1.9%)	16 (3.0%)	0.13
- others	43 (3.4%)	56 (10.6%)	< 0.01

NVP: nevirapine; EFV: efavirenz, NRTI: nucleoside reverse transcriptase inhibitor

All patients developed at least one NNRTI-RAM regardless of EFV and NVP use. The maximal number of NNRTI-RAMs detected per patient was seven. For patients that failed NVP, 25.4% had 1 NNRTI-RAM, 36.9% had 2 NNRTI-RAMs, 27.1% had 3 NNRTI-RAMs, 8.1% had 4 NNRTI-RAMs, 2.1% had 5 NNRTI-RAMs, 0.4% had 6 NNRTI-RAMs, and 0.1% had 7 NNRTI-RAMs. For patients that failed EFV, 13.1% had 1 NNRTI-RAM, 35.8% had 2 NNRTI-RAMs, 30.1% had 3 NNRTI-RAMs, 13.6% had 4 NNRTI-RAMs, 5.7% had 5 NNRTI-RAMs, 1.5% had 6 NNRTI-RAMs, and 0.2% had 7 NNRTI-RAMs (S1 Fig). There was significantly high number of patients that failed NVP-based regimen with 1 NNRTI-RAM compared to those who failed the EFV-based regimen (25.4% vs. 13.1%, p<0.01). There were no significant differences between the patients that failed NVP- and EFV-based regimen with 2, 3, and 7 NNRTI-RAMs. However, there were significantly more patients in the failed EFV-based regimen that had 4 NNRTI-RAMs (p<0.01), 5 NNRTI-RAMs (p<0.01), and 6 NNRTI-RAMs (p = 0.04) compared to the patients that failed the NVP-based regimen. A total of 59 NNRTI-RAMs were identified based on the HIV Drug Resistance Database (Stanford University, U.S.A.) (S2 Fig). Overall, the top 10 most common NNRTI-RAMs found in this study were V90I, followed by A98G, K101E, K103N, V108I, Y181C, M184I, Y188L, G190A and H221Y (Fig 1; listed according to the most frequently detected mutation to the least). Interestingly, the most prevalent NNRTI-RAM E138K reported in the ECHO and THRIVE studies were rarely detected in this study. The authors observed only 2 patients (0.4%) that failed EFV-based regimen had the E138K mutation and none from the failed NVP-based regimen (p = 0.03).

It should be noted that the mutations from the patients that failed NVP-based regimen were different compared to the patients that failed the EFV-based regimen. For example, Y181C (52.7%) was the most common NNRTI-RAM detected in patients that failed NVP-based regimen compared to those that failed EFV-based regimen (14.0%; p<0.01). Another most common NNRTI-RAM detected in patients that failed EFV-based regimen was K103N (61.2%) compared to those that failed NVP-based regimen (18.5%; p<0.01).

**Fig 2:**
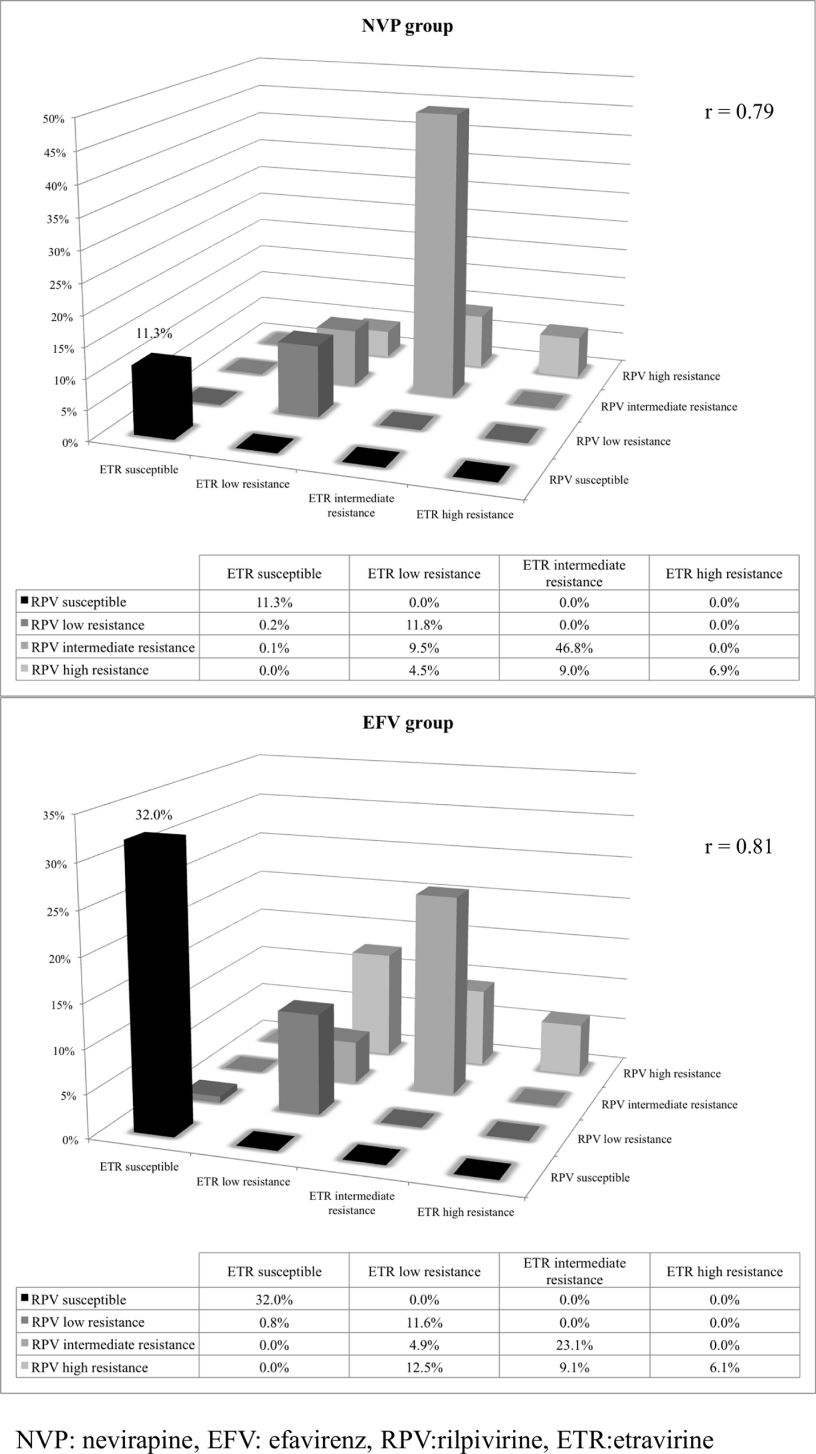
Susceptibility pattern of etravirine and rilpivirine in patients who failed nevirapine and efavirenz-based regimens.

Aside from the mutation profiles, the susceptibilities of ETR and RPV were significantly different in patients who failed NVP- and EFV-based regimen. The overall susceptibilities of ETR (11.5%) and RPV (11.3%) were very low for patients that failed the NVP-based regimens. 144 (11.3%) patients remained susceptible to both ETR and RPV whereas 3 (0.3%) were susceptible to only ETR among those who failed NVP-based regimen (Table 2, Fig 2). Those who were resistant to ETR were not susceptible to RPV. A large proportion of the patients (802 patients, 62.7%) developed intermediate-to-high-level resistance to both ETR and RPV after failing an NVP-based regimen. Among these, 88 (6.9%) patients developed high-level resistance to both ETR and RPV (Table 2, Fig 2).

**Table 2:** Number of patients susceptible to etravirine and rilpivirine.

Group	ETR susceptible	ETR low resistance	ETR intermediate resistance	ETR high resistance	Total
**Failed NVP-based regimen**					
RPV susceptible*	144 (11.3%)	0 (0%)	0 (0%)	0 (0%)	144 (11.3%)
RPV low resistance*	2 (0.2%)	151 (11.8%)	0 (0%)	0 (0%)	153 (12%)
RPV intermediate resistance*	1 (0.1%)	121 (9.5%)	599 (46.8%)	0 (0%)	721 (56.4%)
RPV high resistance*	0 (0%)	58 (4.5%)	115 (9%)	88 (6.9%)	261 (20.4%)
Total*	147 (11.5%)	330 (25.8%)	714 (55.8%)	88 (6.9%)	1279 (100%)
**Failed EFV-based regimen**					
RPV susceptible**	169 (32%)	0 (0%)	0 (0%)	0 (0%)	169 (32%)
RPV low resistance**	4 (0.8%)	61 (11.6%)	0 (0%)	0 (0%)	65 (12.3%)
RPV intermediate resistance**	0 (0%)	26 (4.9%)	122 (23.1%)	0 (0%)	148 (28%)
RPV high resistance**	0 (0%)	66 (12.5%)	48 (9.1%)	32 (6.1%)	146 (27.7%)
Total**	173 (32.8%)	153 (29%)	170 (32.2%)	32 (6.1%)	528 (100%)

NVP: nevirapine; EFV: efavirenz; RPV:rilpivirine, ETR:etravirine

* Pearson's correlation coefficient, NVP group, r = 0.78964306

** Pearson's correlation coefficient, EFV group, r = 0.81354278

As for patients with EFV failure, 169 (32.0%) patients remained susceptible to both ETR and RPV (Table 2, Fig 2). The susceptibilities of ETR (32.8%) and RPV (32.0%) were 3-folds higher (p<0.001) in patients that failed EFV-based regimens compared to those that failed NVP-based regimens as shown in Fig 2. Overall, 202 (38.3%) EFV-resistant patients had intermediate-to-high-level of resistance to ETR and RPV, whereas another 32 (6.1%) EFV-resistant patients had high-level of resistance to both ETR and RPV (Table 2, Fig 2).

## Discussion

The results from the START study [18] will propel many resource-limited countries to start scaling up their ART services. As a result of this, we can also expect a number of patients who will fail the first-line ART regimens with time and would require the second-line ART regimens. In Thailand, we have scaled up ART services at least a year prior to the results reported from the START study and have already seen a gradual increase in the number of patients that need to use the second-line ART regimens. Therefore we know the importance of the construction of the first-line ART regimens because it can significantly and subsequently impact the effectiveness of the second-line ART regimen. Not only that, but the potential cost of the second-line ART regimen should also be contemplated when initially constructing the first-line ART regimens. For example, second-line agent ETR costs more than US$32 when compared to RPV which costs only US$7 per month.

So if RPV can be used strategically as a second-line ARV, this would be highly beneficial to many resource-limited countries because nowadays, the costs of ARVs tend to dictate how many people will be treated and maintained on ART as well as those who will have access to second-line ART regimen. Hence, it is very important to make the most use out of the currently available effective antiretroviral drugs, including utilizing various strategic means, to make the second-line regimen more financially affordable. In order to make this possible, it needs to start with the use of the first-line regimen to ensure that it will have the least negative impact to what ARVs will be available for use as the second-line regimen.

In Thailand[2] and elsewhere in low- and middle-income countries[3], EFV is the most preferred NNRTI for the first-line regimen because of its potency and price. But the problem with the first-generation NNRTI is its low-genetic barrier and broad cross-resistance so other choices need to be seriously considered when the patients are intolerant and/or resistant to EFV or NVP such as ETR. Yet ETR has its own caveat and can be impractical and unfeasible in resource-limited countries because it is 4.6 times more expensive than RPV.

As a result of this, we explored the role of RPV whether it can be safely used in patients that have failed EFV- or NVP-based regimens. Most of our patients in this study have received the backbone that is commonly used in resource-limited setting: d4T+3TC and AZT+3TC or in minority of the patients, TDF+ 3TC. We next examined the mutation profile among patients with first-line regimen failure and observed that most of the patients had thymidine analogue mutations (TAMs) with or without M184V and NNRTI RAMs[19]. In our study, 60.8% and 79.2% were TAM and M184V, respectively.

The most common mutation for RPV in patients who failed NVP-based regimen, compared to the EFV-based regimen, was Y181C (52.7% vs. 14.0%). This particular nonpolymorphic mutation, Y181C, is one of the worst NNRTI-RAM[9,20,21] and it has an intermediate-level resistance to both ETR and RPV. Y181C is notoriously well-known for conferring a 5-fold reduction in susceptibility to ETR[20] and a 3-fold reduction in susceptibility to RPV[9]. Moreover, in combination with other mutations such as V90I, V106I, V179F, G190ASCVT and H221Y, it can synergistically exacerbate the resistance of ETR and RPV.

On the other hand, K103N or K103S, a potential mutation induced by EFV, has no effect on the susceptibility of ETR and RPV. Our results highlight the advantage of EFV over NVP because it can be used with any second generation NNRTI such as ETR or RPV as the subsequent ART regimen.

Another mutation that was investigated was the E138K because it was reported to be 77% prevalent in RPV-failed patients from the ECHO and THRIVE studies[6]. However, this mutation is rarely seen in routine clinical resistant setting (<1%)[22] as reported by observational cohorts from Thailand, Spain and Germany [16,23]. Likewise, our study detected only 0.4% of patients that have failed the first-line NNRTI regimen with E138K. Interestingly, this suggested the different patterns of resistance-associated mutations in non-subtype B viruses.

When we analyzed the data according to the EFV-treatment failure regimen, 32% of the patients were still susceptible to RPV whereas only 11% of the patients who failed NVP-based regimen remained susceptible to RPV. This indicated that RPV can be used as an alternative, second-line antiretroviral agent for EFV-resistant patients. In resource-limited countries, this will be very cost-effective and for the patients, a lot easier to take because of its once-a-day administration and fewer side effects.

Aside from its once-daily administration, its efficacy was found to be similar to EFV. In a dose finding study, 25 mg of RPV (TMC278-C204) was able to maintain viral suppression which was comparable to EFV over 96 weeks with fewer CNS adverse events, cutaneous eruptions and changes in the lipid levels. On top of that, our results suggest that RPV can be used in patients that have early failure of EFV-based regimen[24] in resource-limited countries that uses EFV as its first-line ART regimen[3].

Also, RPV can be combined with other antiretrovirals to construct a potent second-line or third-line regimen. However, there is limited clinical data to support this assumption. Additional prospective studies evaluating the efficacy of RPV in patients who have failed EFV or NPV is warranted to validate the clinical benefits of RPV, particularly in resource-limited settings where RPV can be accessed at a lower cost. However, the use of RPV should be limited in patients with HIV-RNA less than 100,000 copies/mL.

Be that as it may, there are some limitations of the study in ascertaining whether the susceptibility of ETR and RPV are from only one or a sum of resistant mutations. It is possible that patients who have a lot of resistant mutations may have been using the failed regimen for a longer duration compared to those with a shorter duration use of the failed NNRTI regimen but for some patients, we were unable to obtain this information. Also, the genotype tests were not utilized after failure of EFV- and NVP-based regimens which may yield more patients who are susceptible to ETR or RPV. The strategy of sequencing NNRTIs absolutely requires the availability of viral load monitoring and resistance testing that should be implemented in resource-limited settings.

In summary, approximately one-third of HIV-infected Thai patients who failed the EFV-based regimen were fully susceptible to RPV. Compared to ETR, RPV is more cost-effective and can be used as the other alternative second-line ARV agent. However, the early detection of NNRTI failure is very important to preserve activity of RPV in the subsequent regimen.

## Supporting Information

S1 Fig The number of NNRTI-RAMs found in patients who failed either the NVP-or EFV–based regimen. (TIFF)

S2 Fig Distribution of NNRTI-RAMs found in patients who failed either the NVP-or EFV-based regimen. (TIFF)

## References

[1] Adolescents PoAGfAa (2015) Guidelines for the use of antiretroviral agents in HIV-1-infected adults and adolescents. Department of Health and Human Services.

[2] Manosuthi W, Ongwandee S, Bhakeecheep S, Leechawengwongs M, Ruxrungtham K, Phanuphak P, et al (2015) Guidelines for antiretroviral therapy in HIV-1 infected adults and adolescents 2014, Thailand. AIDS Res Ther 12: 12 10.1186/s12981-015-0053-z 25908935 PMC4407333

[3] (2013) Consolidated Guidelines on the Use of Antiretroviral Drugs for Treating and Preventing HIV Infection: Recommendations for a Public Health Approach Geneva: World Health Organization 24716260

[4] Schrijvers R, Desimmie BA, Debyser Z (2011) Rilpivirine: a step forward in tailored HIV treatment. Lancet 378: 201–203. 10.1016/S0140-6736(11)60992-6 21763920

[5] James C, Preininger L, Sweet M (2012) Rilpivirine: a second-generation nonnucleoside reverse transcriptase inhibitor. Am J Health Syst Pharm 69: 857–861. 10.2146/ajhp110395 22555080

[6] Cohen CJ, Andrade-Villanueva J, Clotet B, Fourie J, Johnson MA, Ruxrungtham K, et al (2011) Rilpivirine versus efavirenz with two background nucleoside or nucleotide reverse transcriptase inhibitors in treatment-naive adults infected with HIV-1 (THRIVE): a phase 3, randomised, non-inferiority trial. Lancet 378: 229–237. 10.1016/S0140-6736(11)60983-5 21763935

[7] Molina JM, Cahn P, Grinsztejn B, Lazzarin A, Mills A, Saag M, et al (2011) Rilpivirine versus efavirenz with tenofovir and emtricitabine in treatment-naive adults infected with HIV-1 (ECHO): a phase 3 randomised double-blind active-controlled trial. Lancet 378: 238–246. 10.1016/S0140-6736(11)60936-7 21763936

[8] Imaz A, Podzamczer D (2012) The role of rilpivirine in clinical practice: strengths and weaknesses of the new nonnucleoside reverse transcriptase inhibitor for HIV therapy. AIDS Rev 14: 268–278. 23258301

[9] Rimsky L, Vingerhoets J, Van Eygen V, Eron J, Clotet B, Hoogstoel A, et al (2012) Genotypic and phenotypic characterization of HIV-1 isolates obtained from patients on rilpivirine therapy experiencing virologic failure in the phase 3 ECHO and THRIVE studies: 48-week analysis. J Acquir Immune Defic Syndr 59: 39–46. 10.1097/QAI.0b013e31823df4da 22067667

[10] Sharma M, Saravolatz LD (2013) Rilpivirine: a new non-nucleoside reverse transcriptase inhibitor. J Antimicrob Chemother 68: 250–256. 10.1093/jac/dks404 23099850

[11] Porter DP, Kulkarni R, Fralich T, Miller MD, White KL (2014) Characterization of HIV-1 Drug Resistance Development Through Week 48 in Antiretroviral Naive Subjects on Rilpivirine/Emtricitabine/Tenofovir DF or Efavirenz/Emtricitabine/Tenofovir DF in the STaR Study (GS-US-264-0110). J Acquir Immune Defic Syndr 65: 318–326. 10.1097/QAI.0000000000000017 24525469

[12] Katlama C, Clotet B, Mills A, Trottier B, Molina JM, Grinsztejn B, et al (2010) Efficacy and safety of etravirine at week 96 in treatment-experienced HIV type-1-infected patients in the DUET-1 and DUET-2 trials. Antivir Ther 15: 1045–1052. 10.3851/IMP1662 21041921

[13] Tambuyzer L, Vingerhoets J, Azijn H, Daems B, Nijs S, de Bethune MP, et al (2010) Characterization of genotypic and phenotypic changes in HIV-1-infected patients with virologic failure on an etravirine-containing regimen in the DUET-1 and DUET-2 clinical studies. AIDS Res Hum Retroviruses 26: 1197–1205. 10.1089/aid.2009.0302 20854144

[14] WHO ResNet i-JM, Barcarolo J, Parkin N, de Oliveira T (analysis support), Bertagnolio S. (2012) WHO HIV drug resistance report 2012. WHO report: World Health Organization (WHO).

[15] Zhang F, Dou Z, Ma Y, Zhao Y, Liu Z, Bulterys M, et al (2009) Five-year outcomes of the China National Free Antiretroviral Treatment Program. Ann Intern Med 151: 241–251, W-252. 19687491 10.7326/0003-4819-151-4-200908180-00006

[16] Bunupuradah T, Ananworanich J, Chetchotisakd P, Kantipong P, Jirajariyavej S, Sirivichayakul S, et al (2011) Etravirine and rilpivirine resistance in HIV-1 subtype CRF01_AE-infected adults failing non-nucleoside reverse transcriptase inhibitor-based regimens. Antivir Ther 16: 1113–1121. 10.3851/IMP1906 22024527

[17] Tang MW, Liu TF, Shafer RW (2012) The HIVdb system for HIV-1 genotypic resistance interpretation. Intervirology 55: 98–101. 10.1159/000331998 22286876 PMC7068798

[18] Group ISS, Lundgren JD, Babiker AG, Gordin F, Emery S, Grund B, et al (2015) Initiation of Antiretroviral Therapy in Early Asymptomatic HIV Infection. N Engl J Med 373: 795–807. 10.1056/NEJMoa1506816 26192873 PMC4569751

[19] Sungkanuparph S, Manosuthi W, Kiertiburanakul S, Piyavong B, Chumpathat N, Chantratita W (2007) Options for a second-line antiretroviral regimen for HIV type 1-infected patients whose initial regimen of a fixed-dose combination of stavudine, lamivudine, and nevirapine fails. Clin Infect Dis 44: 447–452. 17205457 10.1086/510745

[20] Vingerhoets J, Tambuyzer L, Azijn H, Hoogstoel A, Nijs S, Peeters M, et al (2010) Resistance profile of etravirine: combined analysis of baseline genotypic and phenotypic data from the randomized, controlled Phase III clinical studies. AIDS 24: 503–514. 10.1097/QAD.0b013e32833677ac 20051805

[21] Rhee SY, Liu T, Ravela J, Gonzales MJ, Shafer RW (2004) Distribution of human immunodeficiency virus type 1 protease and reverse transcriptase mutation patterns in 4,183 persons undergoing genotypic resistance testing. Antimicrob Agents Chemother 48: 3122–3126. 15273130 10.1128/AAC.48.8.3122-3126.2004 PMC478552

[22] Picchio G, Vingerhoets J, Tambuyzer L, Coakley E, Haddad M, Witek J (2011) Short communication prevalence of susceptibility to etravirine by genotype and phenotype in samples received for routine HIV type 1 resistance testing in the United States. AIDS Res Hum Retroviruses 27: 1271–1275. 10.1089/aid.2011.0049 21557669

[23] Anta L, Llibre JM, Poveda E, Blanco JL, Alvarez M, Perez-Elias MJ, et al (2013) Rilpivirine resistance mutations in HIV patients failing non-nucleoside reverse transcriptase inhibitor-based therapies. AIDS 27: 81–85. 10.1097/QAD.0b013e3283584500 22842995

[24] Sungkanuparph S, Manosuthi W, Kiertiburanakul S, Piyavong B, Chantratita W (2008) Evaluating the role of etravirine in the second-line antiretroviral therapy after failing an initial non-nucleoside reverse transcriptase inhibitor-based regimen in a resource-limited setting. Curr HIV Res 6: 474–476. 18855659 10.2174/157016208785861230

