# Supplementary figures and images for: Role of Rilpivirine and Etravirine in Efavirenz and Nevirapine-Based Regimens Failure in a Resource-Limited Country: A Cross- Sectional Study
Source: PLoS One. 2016 Apr 27;11(4):e0154221. doi: 10.1371/journal.pone.0154221 (PMC4847912; doi:10.1371/journal.pone.0154221)

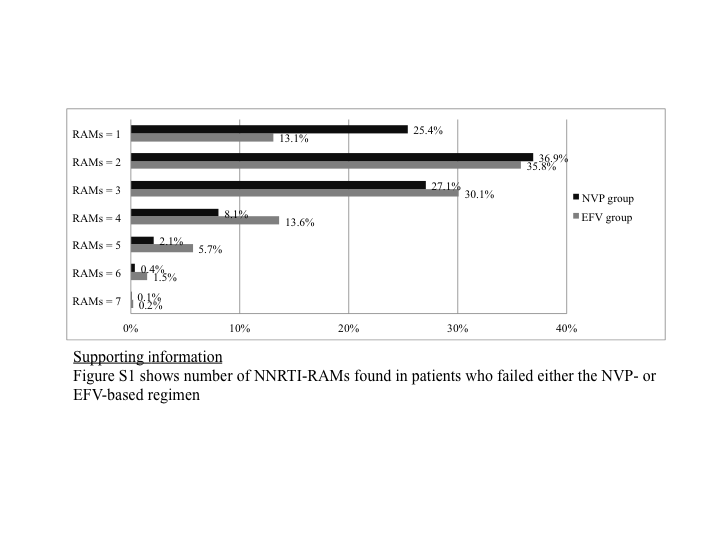

Supplement: S1 Fig — (TIFF) [file pone.0154221.s001.tiff]

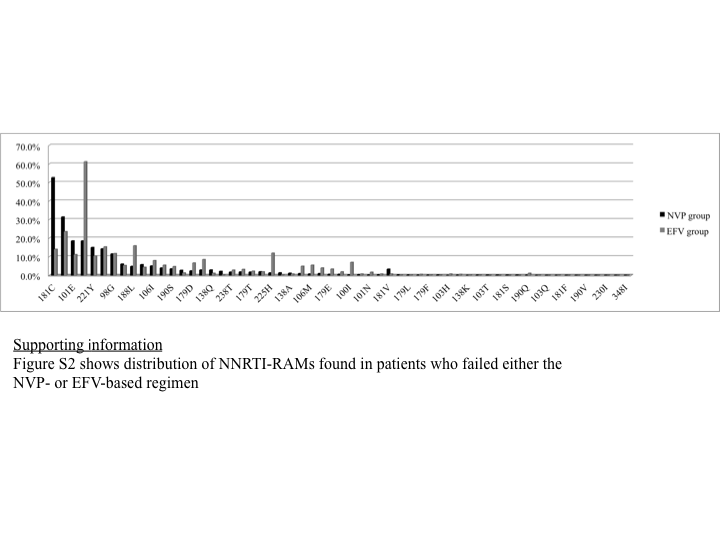

Supplement: S2 Fig — (TIFF) [file pone.0154221.s002.tiff]
